# Swimmer arm-to-shoulder test for early differentiation between shoulder and cervical spine pathology in patients with shoulder pain

**DOI:** 10.1186/s12891-024-08013-9

**Published:** 2024-11-21

**Authors:** Hesham Hamoud, Hany Aly, Yasser A. Elmotaleb, Mohamad M. Ghit, Ahmad Mosalam, Tarek M. Nasrallah, Saad M. El Zokm, Ibrahim Fawzy, Abdelwahab N. Bayoumy, Maha S. Mohamed, Seham A. Elazab, Amal M. Elmesiry, Eman A. Rageh, Mai A. Moussa, Ahmed Elyasaki, Sherif Refaat, Ahmed M. Elhilasy, Ahmed M. El deeb, Walid Elshaitany, Ashraf Eltabiey

**Affiliations:** 1 Rheumatology and Rehabilitation Department, Al-Azhar University, Cairo, Egypt; 2 Rheumatology and Rehabilitation Department, Al-Azhar University, Assiut, Egypt; 3 Rheumatology and Rehabilitation Department, Al-Azhar University, Damietta, Egypt; 4 Physical Medicine and Rehabilitation Department, Ain Shams University, Cairo, Egypt; 5 Rheumatology and Rehabilitation Department, Mansoura University, Mansoura, Egypt; 6 Radiology Department, Ministry of Health, Cairo, Egypt; 7 Radiology Department, Al-Azhar University, Cairo, Egypt; 8 Orthopedic Department, Benha Scientific Hospital, Benha, Egypt; 9 Ashraf Eltabiey, Orthopedic Department, Al-Azhar University, Cairo, Egypt

**Keywords:** Shoulder pain, Cervical spine, Provocative tests, Shoulder impingement

## Abstract

**Background:**

Several tests have been suggested for screening and diagnosis of cervical spine and shoulder girdle conditions underlying shoulder pain with variable degrees of clinical accuracy. The present study aimed to test the reliability, clinical benefit and screening value of the Swimmer Arm-to-Shoulder (SAS) test; a new clinical test developed to differentiate shoulder impingement from cervical radiculopathy in patients with shoulder pain of ≤ 12 weeks.

**Methods:**

The study included 718 patients aged 40–65 years, with unilateral and localized shoulder girdle pain lasting for ≤ 12 weeks. Diagnosis based on clinical, electromyography and radiological findings was considered as the reference gold standard for test assessment.

**Results:**

Clinical diagnosis identified shoulder pathology in 288 patients (40.1%) and cervical spine pathology in 430 patients (59.9%). SAS test was positive in 274 patients (38.2%). The SAS test proved to be effective in distinguishing shoulder from cervical spine pathology with a sensitivity of 89.2% (95% CI: 85.0-92.6%), specificity of 96.1% (95% CI: 93.8–97.7%), PPV of 93.8% (95% CI: 90.5–96.0%), NPV of 93.0% (95% CI: 90.5–94.9%), LR + of 22.6% (95% CI: 14.1–36.0%), LR- of 0.11 (95% CI: 0.08–0.16) and accuracy of 93.3% (95% CI: 91.2–95.0%).

**Conclusions:**

SAS test is an easy to perform, patient dependent and reliable as a screening test and diagnosis confirmatory test.

## Introduction

Shoulder pain is one of the most commonly reported musculoskeletal complaints in clinical practice with a yearly incidence ranging from 7.7 to 62.0 per 1000 persons [1]. The condition is more prevalent among physically active workers beyond the age of 50 [2] and is associated with substantial physical and emotional burden [3]. Pathologically, shoulder pain is characterized by allodynia, impaired conditioned pain modulation and mechanical hyperalgesia [4].

Unfortunately, diagnosis of the musculoskeletal pathology underlying shoulder pain is not simple. In many instances, cervical spine disorders can present with shoulder pain making the differentiation from shoulder conditions clinically challenging [5, 6]. Moreover, it was reported that 35.0% of patients with shoulder impingement syndrome have ipsilateral cervical root compression [7]. In spite of this fact, screening of cervical spine is frequently overlooked in clinical assessment of shoulder pain [8].

Considering the lack of consensus on management of shoulder pain [9] of various etiologies, it’s strongly advocated for patients to have stepwise and evidence-based diagnostic approach involving thorough history taking, detailed clinical examination and radiological studies to establish a definite diagnosis [10, 11] and avoid the risk of having ineffective or misguided interventions [8].

Clinical testing is an essential element of shoulder examination [12]. Several tests have been suggested for screening and diagnosis of cervical spine and shoulder girdle conditions underlying shoulder pain with variable degrees of clinical accuracy [13, 14]. Generally, tests with low sensitivity, high specificity and high likelihood ratio (≥ 2.0) can help to confirm the diagnosis if they are positive while tests with high sensitivity and low likelihood ratio (≤ 0.5) can be used as screening tests [15, 16]. The present study aimed to test the reliability, clinical benefit and screening value of the Swimmer Arm-to-Shoulder (SAS) test; a new clinical test developed to differentiate shoulder impingement from cervical radiculopathy in patients with shoulder pain of ≤ 12 weeks.

## Materials and methods

### Setting and recruitment

The present prospective study was conducted at the outpatient clinics of rheumatology and rehabilitation departments, from January, 2018 through December, 2021. The study protocol was approved by the institutional review board and informed written consent was obtained from all participants before the study in line with the recommendations of Helsinki Declaration on clinical research involving human subjects.

The study included 718 consecutive patients aged 40–65 years, with unilateral and localized shoulder girdle pain lasting for ≤ 12 weeks who were examined and diagnosed to have shoulder or cervical spine pathology according the reference gold standard mentioned below. Patients were excluded if they received any systemic or local treatment for the condition or if they had previous traumatic injuries to the shoulder or the cervical spine, shoulder instability, os acromiale, suprascapular nerve entrapment or systemic inflammatory, autoimmune conditions (e.g. rheumatoid and negative arthritis, systemic lupus erythematosus, scleroderma, etc.) Patients were also excluded if they had cognitive impairment. Patients were also excluded if no definite clinical diagnosis could be concluded or if they had concomitant cervical spine and shoulder pathologies.

### Test technique and development

**Fig. 1:**
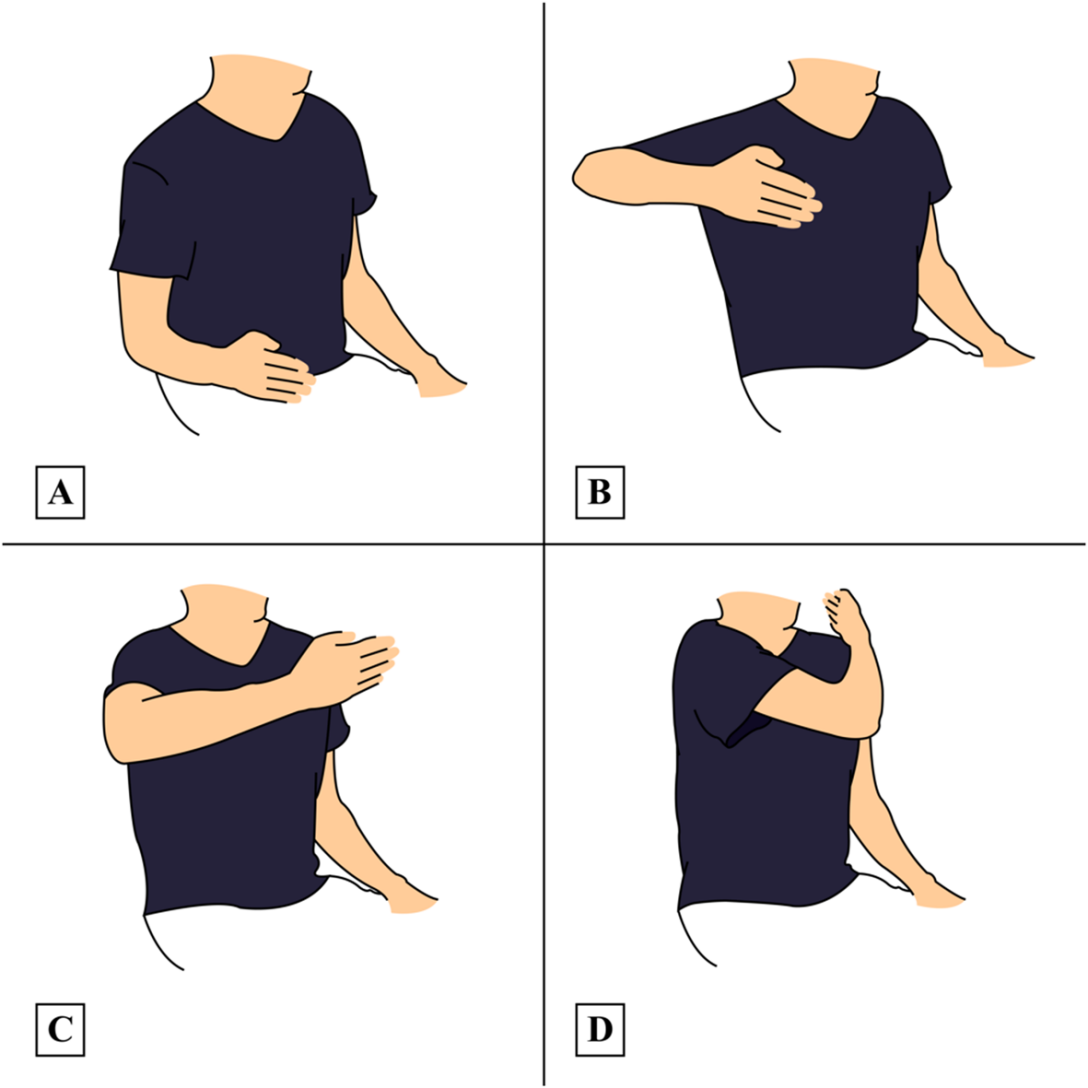
Steps of the swimmer arm-to-shoulder test: **A **Starting position (arm beside the torso with elbow flexion 90^○^), **B** Arm Abduction 90^○^, **C **Swimmer strike, **D **Shoulder touch with hypothenar eminence

With the patient setting and the neutral-position elbow flexed at 90^○^ (Fig. 1A), the affected arm is abducted at 90^○^ (Fig. 1B) followed by horizontal adduction of the shoulder (swimmer strike) (Fig. 1C) to touch the opposite shoulder with the hypothenar eminence of the hand (Fig. 1D). The test is considered positive if the maneuver resulted in shoulder pain with severity over 3 on a 10-cm visual analog scale (VAS) for pain assessment (0 = no pain and 10 = maximum pain). The test is considered negative if such shoulder pain isn’t felt.

The test was initially assessed on a pilot sample of 50 patients with shoulder pain fulfilling the inclusion criteria and 50 age and sex-matched healthy controls. The test was carefully explained for all participants and they were instructed about the use of VAS. All of them could effectively perform the test according to instructions and the test was positive in 31 patients (62.0%) versus no subjects in the control group (*p* < 0.001).

### Clinical diagnosis

The reference gold standard in the present study is diagnosis based on history taking, clinical findings, provocative clinical testing, radiological findings and electromyography (EMG) and following the evidence-based approach suggested by Bokshan et al. [12]. This approach involves the following steps:

1. Appropriate history taking for a possible shoulder or cervical pathology: with emphasis on suspicious etiology and predisposing factors, pain quality, progression and exaggerating and relieving factors and associated sensory or motor complaints.
2. Clinical examination of shoulder and cervical spine: Shoulder is thoroughly examined for muscle atrophy, scapular protraction, retraction, winging, tenderness, passive and active range of motion, drop arm sign. Provocative tests for shoulder pathology included empty can test and O’Brien’s test. Cervical spine is examined for abnormal position, lordosis, kyphosis, movement and tenderness. Strength and reflex testing are also performed. Provocative maneuvers for cervical spinal pathology included Spurling test and “arm squeeze test.
3. Patients with positive provocative shoulder testing are submitted to imaging studies using plain radiography or magnetic resonance imaging if plain radiography was nondiagnostic.
4. Patients with positive provocative cervical spine testing are submitted to imaging studies using plain radiography. If radiculopathy is suspected, MRI is performed. If MRI wasn’t specific, EMG of muscles supplied by the cervical nerve roots is performed as an adjunctive test if suspicion of cervical radiculopathy is high.

To increase accuracy, diagnosis of patients was judged and agreed by two independent rheumatologists with at least 10 years of clinical experience.

### Test performance

For every patient, test performance was independently supervised by two rheumatologists of the study team who were blinded to the clinical diagnosis. The patient repeated the test twice under the supervision of each rheumatologist. Test results were independently reported and blinding of rheumatologists from each other’s interpretation of the test was arranged and supervised by an independent researcher.

### Statistical analysis

Data obtained from the present study were presented as number and percent or mean and standard deviation (SD). Data were coded to secure blinding of the statistician. Statistical calculations were computed using Medcalc version 22.009 (MedCalc Software Ltd, Ostend, Belgium). Test sensitivity was calculated as number of true positives divided by the sum of numbers of true positives and false negatives while test specificity was calculated as number of true negatives divided by sum of numbers of true negatives false positives. Positive predictive value (PPV) was calculated as number of true positives divided by sum of numbers of true and false positives and negative predictive value (NPV) was calculated as number true negatives divided by sum of numbers of true and false negatives. Positive likelihood ratio (LR+) is calculated as sensitivity divided by 1-specificity while negative likelihood ratio (LR-) is calculated as 1-sensitivity divided by specificity [17]. p value less than 0.05 was considered statistically significant.

## Results

 The present study included 718 patients with shoulder pain. They comprised 258 males (35.9%) and 460 females (64.1%) with an age of 55.3 ± 7.2 years. Clinical diagnosis identified shoulder pathology in 288 patients (40.1%) and cervical spine pathology in 430 patients (59.9%). SAS test was positive in 274 patients (38.2%) (Table 1). The SAS test showed fair agreement with the gold standard diagnosis (Kappa = 0.24, *p* < 0.001) (Table 2).

**Table 1:** Basic findings in the studied patients (*n*=718)

**Age** (years)	
mean ± SD	55.3 ± 7.2
≤ 50	176 (24.5)
> 50	542 (75.5)
**Male/female** n	258/460
**Clinical diagnosis** n (%)	
Shoulder pathology	288 (40.1)
Cervical spine pathology	430 (59.9)
**Positive SAS test** n (%)	274 (38.2)

**Table 2:** Agreement between clinical diagnosis and SAS test results

	Gold standard		Measure of agreement	
	Shoulder +ve *n*=288	Shoulder -ve *n*=430	Kappa	*p* value
SAS				
Shoulder +ve n (%)	257 (89.2)	17 (4.0)	0.24	<0.001
Shoulder -ve n (%)	31 (10.8)	413 (96.0)		

 The SAS test proved to be effective in distinguishing shoulder from cervical spine pathology with a sensitivity of 89.2% (95% CI: 85.0-92.6%), specificity of 96.1% (95% CI: 93.8–97.7%), PPV of 93.8% (95% CI: 90.5–96.0%), NPV of 93.0% (95% CI: 90.5–94.9%), LR + of 22.6% (95% CI: 14.1–36.0%), LR- of 0.11 (95% CI: 0.08–0.16) and accuracy of 93.3% (95% CI: 91.2–95.0%). Test results classified by patients’ age and sex are shown in Table 3.

**Table 3:** Test performance for detection of shoulder pathology

	**All patients** *N*=718	**Age categories **(years)		**Sex categories**	
		≤ 50 (*n*=176)	> 50 (*n*=542)	Male (*n*=258)	Female (*n*=460)
**Sensitivity**	89.2 (85.0-92.6)	83.1 (71.7-91.2)	91.0 (86.5-94.4)	88.1 (79.2-94.1)	89.7 (84.7-93.5)
**Specificity **	96.1 (93.8-97.7)	91.0 (84.1-95.6)	97.8 (95.5-99.1)	97.7 (94.2-99.4)	94.9 (91.5-97.3)
**PPV**	93.8 (90.5-96.0)	84.4 (74.8-90.8)	96.7 (93.3-98.4)	94.9 (87.5-98.0)	93.4 (89.2-96.0)
**NPV**	93.0 (90.5-94.9)	90.2 (84.2-94.0)	94.0 (91.1-96.0)	94.4 (90.5-96.8)	92.1 (88.5-94.6)
**LR+**	22.6 (14.1-36.0)	9.2 (5.1-16.8)	41.5 (19.9-86.4)	38.3 (14.5-101.3)	17.7 (10.4-30.1)
**LR-**	0.11 (0.08-0.16)	0.19 (0.11-0.32)	0.09 (0.06-0.14)	0.12 (0.07-0.22)	0.11 (0.07-0.16)
**Accuracy**	93.3 (91.2-95.0)	88.1 (82.3-92.5)	95.0 (92.8-96.7)	94.6 (91.1-97.0)	92.6 (89.8-94.8)

## Discussion

The present study assessed the performance of a new clinical test designed to differentiate shoulder and cervical spine pathologies in patients with shoulder pain. According to our findings, the Swimmer Arm-to-Shoulder (SAS) test proved to be effective as a screening test and as a diagnosis confirmatory test in all patients and in patients categorized according to age and sex. In comparison to many provocative tests, SAS is totally self-performed by patients without interference of the examiner.

Biomechanically, the test combines multiple movements to test the function of shoulder girdle muscles involved. On arm abduction with flexed elbow, the rotator cuff muscles in addition to the deltoid the major contributors to the abduction torque. However, the supraspinatus is considered the most influential shoulder abductor having more effective moment arm [18]. To make the swimmer’s strike, the arm is internally rotated and adducted thus activating many muscles particularly the subscapularis [19].

The third movement of the test which included touching the opposite shoulder with hypothenar eminence of the affected side provides similar effect to other tests investigating the subscapularis including bear-hug test [20], belly press test [21, 22], belly-off test [23] and liftoff test [24] without the interference of the examiner.

In comparison to SAS test, the belly press has a reported sensitivity and specificity of 28-50% and 96-99% while the belly-off test has a reported sensitivity and specificity of 86% and 91%, the liftoff test has sensitivity and specificity of 12-25% and 95-100% and the bear hug test has sensitivity and specificity of 19-60% and 81-92% [14].

It’s important to emphasize that SAS and other provocative maneuvers can’t replace sophisticated and evidence-based approaches for established of definitive diagnosis on the basis of careful history taking, thorough clinical examination and standard laboratory and imaging studies. Instead, these tests provide clinicians with a readily available and easily performed screening tool that can accelerate or facilitate the demanding process of clinical diagnosis particularly in patients with confusing pathological entities.

## Conclusions

In conclusion, the present study suggests that SAS test is an easy to perform, patient dependent and reliable both as a screening test and diagnosis confirmatory test. The test assesses the function of shoulder girdle muscles in multiple directions and doesn’t require examiner’s intervention with its related bias.

### Limitations

Conclusions of the present study are limited by being a single-center study. Also, test results depend on the subjective perception of pain which is an inherent limitation of clinical tests using subjective symptoms.

## Data Availability

Data will be available from the corresponding author upon reasonable request.
